# Preparation and Sensing Study of *Houttuynia cordata*-Based Carbon Quantum Dots

**DOI:** 10.3390/molecules30183668

**Published:** 2025-09-09

**Authors:** Min Ye, Lifen Meng

**Affiliations:** 1 School of Chemical Engineering, Guizhou University of Engineering Science, Bijie 551700, China; 2 Analytical & Testing Center, Guizhou University of Engineering Science, Bijie 551700, China

**Keywords:** *Houttuynia cordata*-carbon quantum dots (*Hc-CQDs*), fluorescent quenching, biomass, wastewater

## Abstract

This study used Houttuynia cordata as the precursor to prepare high fluorescence quantum yield carbon quantum dots (Hc-CQDs) by a simple hydrothermal method. The surface of the Hc-CQDs contained abundant functional groups, such as carboxyl, hydroxyl, and amino groups, which indicated the Hc-CQDs had good water solubility. On the basis of the excellent fluorescence characteristics of Hc-CQDs, a sensor was constructed to achieve high selectivity detection of Cr^3+^, and the detection limit of the Hc-CQDs was 49 μg/L. The sensor also exhibited strong anti-interference ability and excellent reproducibility, which was used for the determination of Cr^3+^ in environmental water samples, and its spiked recovery rate reached over 90%. Therefore, the Hc-CQDs had potential application in the analysis.

## 1. Introduction

*Houttuynia cordata* is a perennial herb in the genus *Houttuynia* of the family *Houttuyniaceae*. The whole herb can be used medicinally, is cold and pungent in nature, belongs to the lung meridian, and has a special fishy flavor [1]. Its main active constituents include fisetin (decanoylacetaldehyde), the antimicrobial core component, which inhibits a wide range of pathogenic bacteria and viruses. Essential oils have both anti-inflammatory and antiviral effects. Quercetin and flavonoids promote capillary dilation and enhance diuretic and antiallergic effects. Polysaccharides regulate immune function and increase serum protein levels. Other components include potassium, organic acids, etc. [2,3,4]. It can be used to treat many diseases, which makes it a very valuable herb for research [5,6].

Although Cr^3+^ can also be absorbed by the human body as a trace element, it is only a non-essential and highly toxic heavy metal for microorganisms and plants. However, if the human body is exposed to Cr^3+^ for a long time, it can also cause various toxic reactions, including chromium ulcers, allergic dermatitis, liver failure, etc. In severe cases, it may also lead to cancer. Studies have also shown that Cr^3+^ can cause damage to the liver function and metabolic system of animals [7]. Wastewater containing trivalent chromium ions will enter the soil through infiltration and other means when discharged into the environment. Cr^3+^ will be adsorbed by soil particles, resulting in excessive chromium content in the soil. This will change the physical and chemical properties of the soil, reduce soil fertility, affect the activity and community structure of microorganisms in the soil, and ultimately disrupt the balance of the soil ecosystem, which is not conducive to plant growth and development [8,9].

Carbon quantum dots (CQDs) are a class of zero-dimensional carbon nanomaterials consisting of ultrafine, dispersed quasispherical carbon nanoparticles, typically less than 10 nm in size, with remarkable fluorescence and tunable photoluminescence properties [10,11]. Their core structure consists of sp^2^/sp^3^ hybridized carbon with hydroxyl- and carboxyl-rich functional groups on their surfaces, which are both water soluble and chemically stable [12]. It is characterized by its rich surface oxygen-containing functional groups and optical excitation/emission wavelength tunable properties, and a wide range of raw material sources (e.g., *Houttuynia cordata*), which can be directed to modulate its physicochemical properties [13,14]. It has a wide range of applications [15], including in environmental and chemical testing to form a colorimetric-fluorescence dual sensor system, in the detection of pollutants such as glucose and heavy metal ions [16,17], and as a chemical probe for monitoring water quality and air pollution [18,19].

In this work, the *Houttuynia cordata*-based CQDs were prepared by a one-step hydrothermal method, using *Houttuynia cordata* as the carbon source and ethylenediamine as the nitrogen source. The novelty of this research was environmentally friendly, cost-effective, and non-toxic, and compared with traditional organic fluorescent molecules [20], the detection of trace Cr^3+^ under alkaline conditions could be realized by *Hc-CQDs.* The *Hc-CQDs* had strong photostability and good resistance to photobleaching, solving the problem of signal attenuation in the detection of Cr^3+^ in complex environmental water systems.

## 2. Results and Discussion

### 2.1. Optimization of Synthesis Conditions

**Figure 1:**
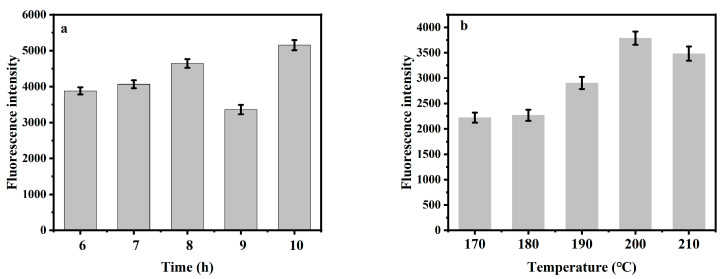
(**a**) The influence of synthesis time and (**b**) the synthesis temperature on fluorescence intensity of *Hc-CQDs* (*n* = 3).

The effects of the synthesis temperature and time on the fluorescence intensity of *Hc-CQDs* were investigated. As shown in Figure 1a,b, the rapid growth of particles, the gradual formation of carbon nuclei, and the increasing number of functional groups on the surface with increasing temperature led to a gradual increase in fluorescence intensity, from which 200 °C was the optimal reaction temperature for the synthesis of *Hc-CQDs*. In addition, the fluorescence intensity of the *Hc-CQDs* reached a maximum when the reaction time was 10 h. As the reaction time continued to increase, the fluorescence intensity of the *Hc-CQDs* decreased, and a longer synthesis time usually led to the aggregation of more carbon atoms into the CQDs [21,22].

### 2.2. Characterization of Hc-CQDs

**Figure 2:**
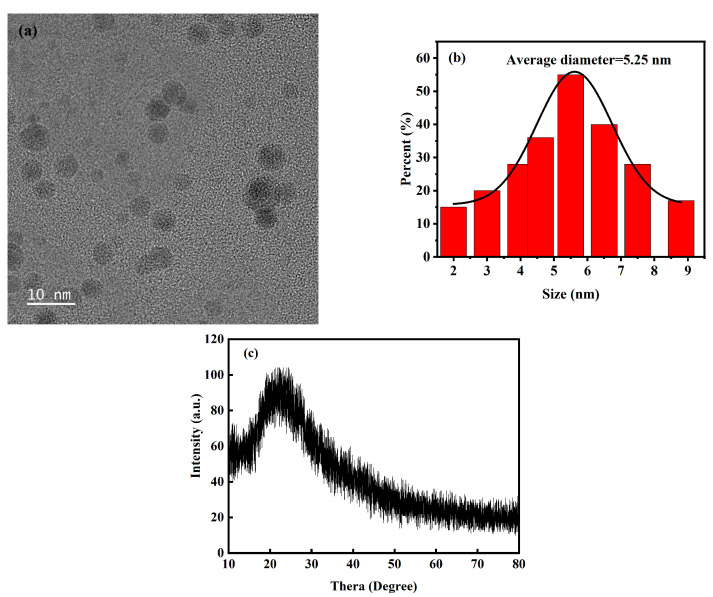
(**a**) TEM image, (**b**) the size distribution, and (**c**) XRD spectrum of *Hc-CQDs*.

The shape and microstructure of the *Hc-CQDs* were characterized via TEM. As shown in Figure 2a, the prepared *Hc-CQDs* were uniform in size and approximately circular, with no obvious aggregation [23]. Using nano measure to calculate the size of 50 samples, Figure 2b shows the statistical distribution of *Hc-CQDs* particle size. From the figure, it could be analyzed that the particle size distribution range of the synthesized *Hc-CQDs* was between 2.0 and 9.0 nm, and the calculated average particle size was 5.25 nm. X-ray diffraction (XRD) analysis of *Hc-CQDs* (Figure 2c) revealed a diffraction peak at 2θ = 23.28°, suggesting that the composition of *Hc-CQDs* was similar to that of graphite and had graphene-like structural features [24,25].

**Figure 3:**
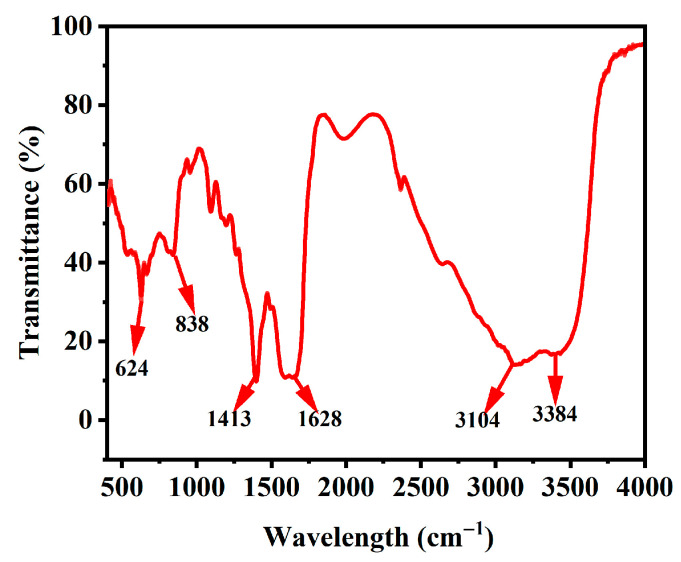
FT-IR spectrum of *Hc-CQDs*.

The surface functional groups of the *Hc-CQDs* were investigated via FT-IR. In Figure 3, the absorption bands at 3384 and 3104 cm^−1^ belong to the O-H stretching vibration. The absorption peak at 1628 cm^−1^ was assigned to C = O bond stretching vibrations, and the peak at 1413 cm^−1^ was attributed to C-N bond stretching vibrations. The absorption peaks at 838 and 624 cm^−1^ were assigned to the asymmetric stretching vibrations of C-O-C bonds. The N atoms were also successfully doped into the *Hc-CQD* skeleton. The results showed that the prepared *Hc-CQDs* had many N- and O-containing functional groups, which were conducive to improving their water solubility [26,27,28].

**Figure 4:**
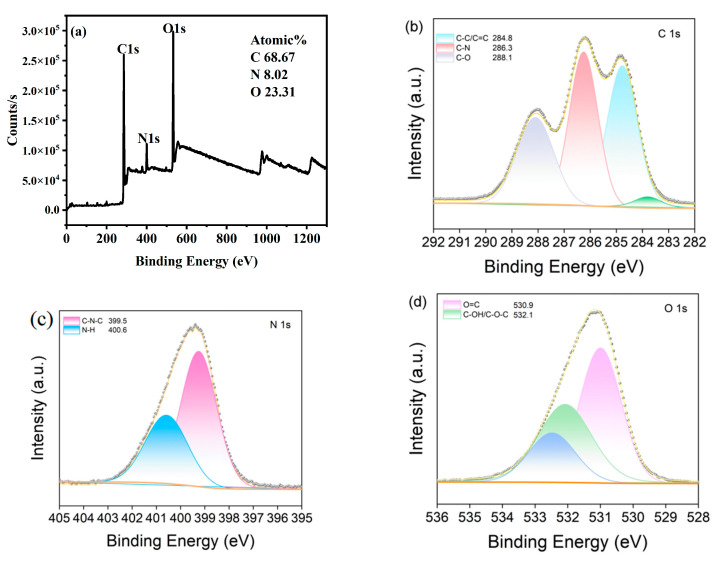
(**a**) XPS survey spectra of *Hc-CQDs*, and high-resolution XPS spectra of C 1s (**b**), N 1s (**c**), O 1s (**d**).

The elemental composition and surface groups of the *Hc-CQDs* were analyzed via XPS. The full XPS spectrum of *Hc-CQDs* in Figure 4a shows three typical peaks located at 286, 400, and 533 eV, which belong to C 1 s, O 1 s, and N 1 s, respectively. The *Hc-CQDs* contained 68.67%, 8.02%, and 23.31% C, O, and N, respectively. XPS and FT-IR results confirmed the successful doping of N into the backbone of *Hc-CQDs* [29,30]. In the high-resolution C 1 s spectrum (Figure 4b), there are four peaks with different binding energies. The peaks at 284.8 eV, 286.3 eV, and 288.1 eV are attributed to C-C/C=C, C-N, and C-O, respectively. In the high-resolution N 1 s spectrum (Figure 4c), the peak with a binding energy of 399.5 eV originates from C-N-C, and the peak at 400.6 eV indicates the presence of N-H functional groups. Figure 4d shows the high-resolution O 1 s spectrum, which contains four characteristic peaks at 530.9 eV and 532.1 eV, corresponding to O=C and C-OH/C-O-C, respectively.

### 2.3. Optical Properties of the Hc-CQDs

**Figure 5:**
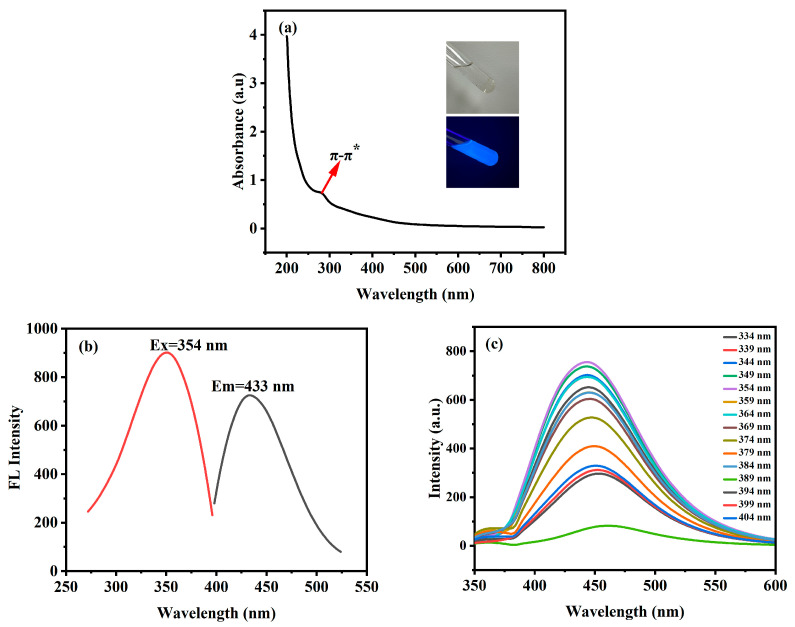
(**a**) UV-Vis (Insert: the image of *Hc-CQDs* under natural light and UV light of 365 nm) and (**b**) fluorescence emission and excitation spectra of *Hc-CQDs*, (**c**) fluorescence emission spectra of *Hc-CQDs* under different excitation wavelengths from 334 to 404 nm.

The optical properties of the *Hc-CQDs* were investigated via a UV-visible spectrophotometer and a fluorescence spectrophotometer. As shown in Figure 5a, *Hc-CQDs* had a UV absorption peak at 280 nm, which corresponded to the π–π* jump, which was usually caused by the chemical structure and energy band structure inside the CQDs. As shown in Figure 5b, the maximum excitation and emission wavelengths of *Hc-CQDs* were 354 nm and 433 nm, respectively. The inset in Figure 5a shows the colors of *Hc-CQD* solutions under natural light and UV lamp (365 nm) irradiation. Under a UV lamp, the *Hc-CQD* solution showed strong blue fluorescence. Using quinine sulfate (QY = 0.54) as a reference [31], the QY of *Hc-CQDs* in water was 12.28%.

As shown in Figure 5c, the maximum emission peak of *Hc-CQDs* was at 433 nm when the excitation wavelength gradually increased from 334 to 404 nm. The fluorescence emission spectra of *Hc-CQDs* gradually shifted toward red, and their emission spectra exhibited tunability. This mechanism might be related to the energy band transition induced by the surface state effect of *Hc-CQDs*.

**Figure 6:**
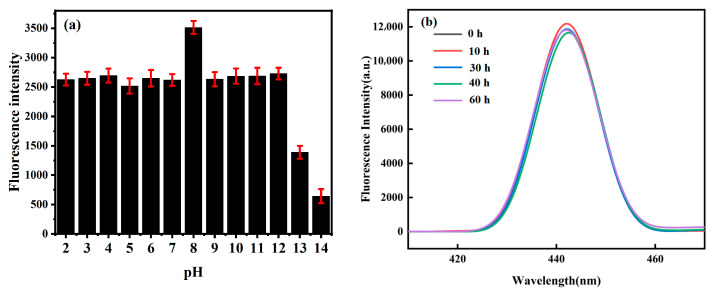
(**a**) Fluorescence intensity of *Hc-CQDs* under different (**a**) pH and (**b**) storage times.

To further evaluate the application prospects of *Hc-CQDs* in optics, the optical stability of *Hc-CQDs* was studied. Figure 6a shows the fluorescence intensity of *Hc-CQDs* in different pH solutions, indicating that the fluorescence intensity of *Hc-CQDs* remained stable under acidic conditions. When the pH of the *Hc-CQD* solution gradually increased to 8, the fluorescence intensity reached its maximum value. As the pH further increased, the fluorescence intensity decreased until the pH reached 13, at which point the fluorescence was significantly quenched. The results indicated that the fluorescence of *Hc-CQDs* was highly sensitive to extremely alkaline pH environments and that *Hc-CQDs* have the potential to be used as sensors in alkaline pH environments.

The *Hc-CQDs* were placed in the refrigerator for 60 h, the fluorescence spectrum was measured every 10 h, and the fluorescence intensity was recorded at the optimal emission point (Figure 6b). The fluorescence intensity of *Hc-CQDs* showed no significant quenching phenomenon, indicating that *Hc-CQDs* had excellent resistance to photobleaching. In summary, the *Hc-CQDs* exhibited good optical stability in addition to pH interference.

### 2.4. Fluorescence Detection of Cr^3+^

#### 2.4.1. Linear Range

**Figure 7:**
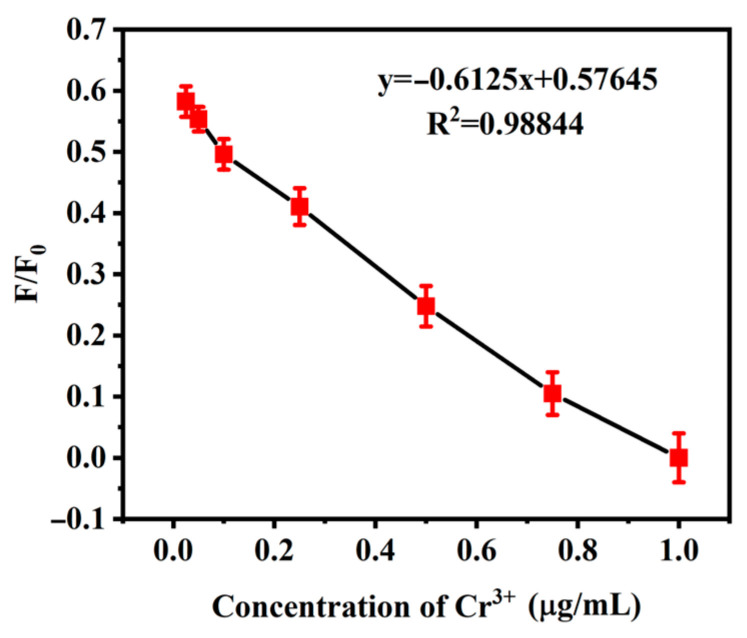
The linear relationship between the fluorescence changes in *Hc-CQDs* and the concentration of Cr^3+^ (F_0_ and F representing fluorescence intensity before and after the addition of Cr^3+^).

Figure 7 shows the quenching curves of *Hc-CQDs* fluorescence at different concentrations of Cr^3+^. The graph shows that *Hc-CQDs* exhibited a good linear correlation in the range of 0.025–1.0 μg/mL. As shown in Figure 7, the linear regression equation for *Hc-CQDs* was y = −0.6125x + 0.576, R^2^ = 0.98844 (where x was the concentration of Cr^3+^). According to the principle of three standard deviations, the limit of detection (LOD) can be calculated as LOD = 3 σ/k. Where σ is the standard deviation of *Hc-CQDs*, and k is the slope of the calibration curve [32]. The LOD of *Hc-CQDs* was calculated to be 49 μg/L. The results indicate that *Hc-CQDs* have good selectivity and a low detection limit for Cr^3+^.

#### 2.4.2. Selectivity of Cr^3+^

**Figure 8:**
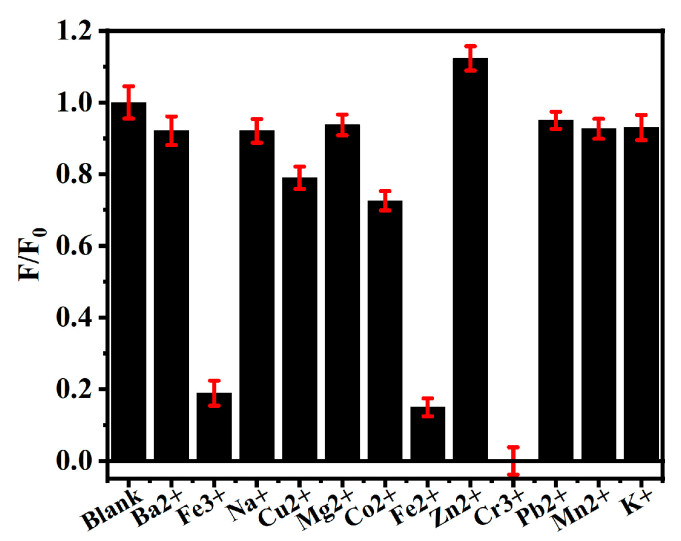
The impact of different metal ions on *Hc-CQDs*.

In this experiment, the selectivity of *Hc-CQD* fluorescent probes for detecting Cr^3+^ was evaluated. The influence of different metal ions on the fluorescence intensity of the *Hc-CQDs* was examined to determine their high selectivity and sensitivity to Cr^3+^. As shown in Figure 8, Cr^3+^ significantly quenched the fluorescence signal of *Hc-CQDs*, indicating that *Hc-CQDs* had high sensitivity to Cr^3+^. However, in the presence of other metal ions, the change in fluorescence of the *Hc-CQDs* was relatively small and could be ignored.

#### 2.4.3. Fluorescence Quenching Mechanism

**Figure 9:**
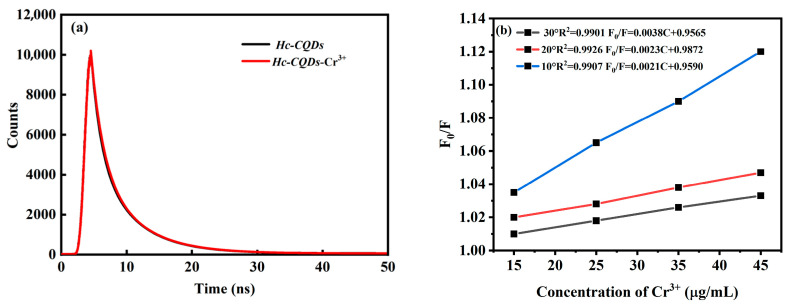
(**a**) Fluorescence emission decay curves of *Hc-CQDs* and *Hc-CQDs*-Cr^3+^, (**b**) Stern–Volmer plots for the *Hc-CQDs*-Cr^3+^ system at three different temperatures.

To investigate the mechanism of fluorescence quenching for the detection of Cr^3+^ via *Hc-CQDs*, we measured the fluorescence lifetimes of *Hc-CQDs* with and without Cr^3+^. The PL decay process of the sample was evaluated via the multidimensional time-correlated single-photon counting (TPSPC) method (Figure 9a). Interestingly, we observed no significant change in the fluorescence lifetime decay curves between *Hc-CQDs*-Cr^3+^ and *Hc-CQDs*, indicating that the addition of Cr^3+^ did not affect the fluorescence lifetime. These results showed that the fluorescence lifetimes of the *Hc-CQDs* and *Hc-CQDs*-Cr^3+^ systems were *τ_0_ =* 4.553 ns and τ = 4.517 ns (*τ_0_/*τ ≈ 1), respectively, indicating that the fluorescence quenching mechanism was static quenching [33]. Furthermore, the dynamic fluorescence quenching constant increased with increasing system temperature, suggesting an improvement in the energy transfer efficiency and an increase in the effective collision of molecules. Conversely, the static fluorescence quenching constant decreased with increasing temperature. Assuming that the mechanism is static quenching, it can be described by the Stern–Volmer equation [34].

*F*_0_/*F* = 1 + *Ksv*[C] = 1 + *K_q_τ*_0_[C]

where *F*_0_ and *F* represent the fluorescence intensities of *Hc-CQDs* at 433 nm with/without Cr^3+^, respectively; *Ksv* represents the Stern–Volmer quenching constant; [C] represents the concentration of the quencher; *K_q_* represents the quenching rate constant; and *τ*_0_ represents the fluorescence lifetime of the *Hc-CQDs*.

The Stern–Volmer curves of Cr^3+^ quenched *Hc-CQDs* at different temperatures were obtained, as shown in Figure 9b. The quenching constants at 10 °C, 20 °C, and 30 °C are 0.0021, 0.0023, and 0.0038, respectively. The quenching constants gradually decrease with increasing temperature, supporting the conclusion that the quenching mechanism is static quenching [35,36].

#### 2.4.4. Detection of Cr^3+^ in Actual Samples

To verify the feasibility and application potential of the developed fluorescent probe in actual samples, Cr^3+^ in environmental water samples was detected to evaluate the performance and reliability of the probe. Tap water, lake water, and river water were selected and pretreated to ensure accuracy and reproducibility in the experiments. Next, known concentrations of Cr^3+^ were added to these pretreated samples to simulate different Cr^3+^ concentrations. Then, the changes in the fluorescence signal of the probe were recorded. As shown in Table 1, when the method developed in this experiment was used, no Cr^3+^ was detected in the actual samples. The recovery rate of Cr^3+^ in the water sample was between 90% and 95%, and the relative standard deviation (RSD%) was between 1.34% and 2.18%. The results indicated the feasibility of the developed fluorescent probe in actual samples, and *Hc-CQDs* could be used as effective fluorescent probes with high sensitivity and selectivity for detecting Cr^3+^ in actual samples (Table 2).

## 3. Experimental Section

### 3.1. Reagents and Instruments

*Houttuynia cordata* were purchased from a local farmer’s market and were fresh. Ethylenediamine was purchased from Shanghai Aladdin Biochemical Technology Co. (Shanghai, China) Ferric chloride, sodium nitrate, copper sulfate, anhydrous magnesium sulfate, cobalt acetate, ferrous chloride, zinc sulfate heptahydrate, chromium trichloride, lead acetate, manganese acetate, potassium nitrate, and barium nitrate were purchased from Shanghai Mack Biochemistry Technology Co., (Shanghai, China).

A fluorescence spectrophotometer (Metash, F97PRO, Shanghai, China), ultraviolet—visible spectrophotometer (Metash, UV-5200PC, Shanghai, China), transmission electron microscope (TEM, JEOL JEM-F200, Tokyo, Japan), X-ray photoelectron spectrometer (USA, XPS, Thermo Scientific K-AlpHa), X-ray diffractometer (XRD, HAOYUAN DX-2700BH, Shanghai, China), Fourier transform infrared spectrometer (FTIR, WQF-530A, Shanghai, China) and steady-state/transient fluorescence spectrometer (PL, Edinburgh FLS1000, Edinburgh, UK) were used for this experiment.

### 3.2. Preparation of Houttuynia Cordata-Based CQDs

Weigh 3 g of *Houttuynia cordata* powder and 1 g of ethylenediamine, 75 mL of distilled water was added, the mixture was stirred with a magnetic stirrer for 30 min, and then the mixture was poured into the polytetrafluoroethylene high-pressure reactor at 200 °C for 10 h to prepare the *Hc-CQDs*. Then, the reaction mixture was filtered to remove solid particles and subsequently dialysed with a 3000 Da dialysis bag for 48 h. Finally, black *Hc-CQDs* powder was obtained by freeze-drying.

### 3.3. Preparation of Characterization Samples for Hc-CQDs

The FTIR is measured by a Fourier transform infrared spectrometer using the commonly used compression method in infrared measurement. The solid sample is mixed with KBr powder in a ratio of 1:100, and then the mixed sample is thoroughly ground and evenly mixed. It is then loaded into a compression mold and pressed into shape. The Fourier transform infrared spectrometer for measuring samples has a resolution of 4 cm^−1^, with 20 sample scans and a scanning range of 400 cm^−1^ to 4000 cm^−1^.

TEM: Take 5 g of *Hc-CQDs* powder and disperse it in an ethanol solution for ultrasonic treatment. Then, take a few drops of the dispersed *Hc-CQDs* liquid and add them dropwise onto a copper mesh. After drying, accelerate the voltage to 200 KV and take photos of the morphology (high-resolution), energy spectrum point scan, energy spectrum line scan, diffraction, energy spectrum surface scan, and other test items.

XPS: Set instrument parameters, excitation source settings for X-ray source: AI K α-ray (HV = 1486.6 eV), beam spot: 400 mm, analysis chamber vacuum degree superior to 5.0 × 10^−7^ mBar, working voltage: 12 kv, filament current: 6 mA, full spectrum scan: conduction energy of 100 eV with a step size of 1 eV, narrow spectrum scan: conduction energy of 50 eV with a step size of 0.1 eV. After setting, take *Hc-CQDs* and press them onto a sample disk. Place the sample into the sample chamber of the Thermo Scientific K-Alpha XPS instrument (Waltham, MA, USA). When the pressure in the sample chamber is less than 2.0 × 10^−7^ mbar, send the sample into the analysis chamber.

XRD: Mix and grind the sample with KBr in a ratio of 2–5 mg sample to 100–120 mg KBr, ensuring no obvious particles. Pour the mixture into the mold, vacuum, and apply pressure for a few minutes to form a transparent disc. Fix the powder with a glass sample plate and then scan and analyze it in the instrument.

### 3.4. Detection of Metal Ions by Hc-CQDs

One milliliter of each different metal ion solution was mixed with 40 μL of 0.1 mg/mL *Hc-CQDs* solution in a 10 mL centrifuge tube. Then, 2 mL of acetate buffer solution was added, and the mixture was fixed to scale with distilled water and left to stand for 30 min. The fluorescence intensity was measured with a fluorescence spectrophotometer (Ex = 354 nm, Em = 433 nm).

### 3.5. Analysis of Metal Ions in Real Samples

In the experiment, river water and laboratory tap water were selected as real samples to investigate the effectiveness of the *Hc-CQDs* fluorescent sensor in detecting chromium ions in real samples. Before analysis, real water samples were pretreated. First, a certain number of water samples (laboratory tap water and Liucang River water) were taken and filtered through a 0.22 μm membrane to remove impurities, centrifuged at 3000 r/min for 15 min, and then analyzed as described in Section 3.4.

## 4. Conclusions

This study synthesized a novel green carbon quantum dot (*Hc-CQDs*) based on the biomaterial *Houttuynia cordata* and ethylenediamine via the hydrothermal method. Structural characterization showed that *Hc-CQDs* were spherical, evenly distributed, and had an average size of 5.25 nm. Spectral studies and reactions indicated that *Hc-CQDs* could be used to detect Cr^3+^ in wastewater. *Hc-CQDs*’ surface had abundant hydroxyl, carboxyl groups, and nitrogen atoms, which could emit bright blue fluorescence. After mixing *Hc-CQDs* with Cr^3+^, the reaction between the chromophore and Cr^3+^ led to varying degrees of fluorescence quenching. Therefore, *Hc-CQDs*’ applicability for trace Cr^3+^ detection and treatment in real water samples was demonstrated, which broadened their application in serving as potential nanoprobes in the future.

## Figures and Tables

**Table 1:** *Hc-CQDs* were used to detect Cr^3+^ in water samples (*n* = 3).

Samples	Spiked Concentration (mg/L)	Detected Concentration (mg/L)	Recovery (%)	RSD (%)
Tap water	40	36.8	92.0	1.34
Lake water	50	47.5	95.0	2.18
River water	60	54	90.0	1.59

**Table 2:** The comparison of the analytical performance of *Hc-CQDs* against previously reported optical sensors for Cr^3+^.

Methods	Linear Range	LOD	Actual Sample	References
S/N-B-CQDs	0–0.5 mM	6 mM	Mineral water	[37]
CS	0–700 mM	6.72 mM	-	[38]
Spinach direct juice-CDs	-	0.138 mM	-	[39]
*Hc-CQDs*	0.025–1.0 μg/mL	49 μg/L	Tap water, lake water, and river water	This paper

## Data Availability

No new data were created or analyzed in this study. Data sharing is not applicable to this article.
